# Study protocol: outcomes of REvision surgery after primary JOINt replacement (REJOIN) study: a prospective cohort study

**DOI:** 10.1302/2633-1462.711.BJO-2026-0217

**Published:** 2026-11-09

**Authors:** Douwe Rijntjes, Robert J. P. van der Wal, Suzan H. M. Verdegaal, Taco Gosens, Jakob van Oldenrijk, Tommy S. de Windt, Rudolf W. Poolman, Rob G. H. H. Nelissen, Maaike G. J. Gademan, REJOIN study group

**Affiliations:** 1 Department of Orthopaedics, Leiden University Medical Center, Leiden, the Netherlands; 2 Department of Orthopedics, Alrijne Ziekenhuis, Leiderdorp, the Netherlands; 3 Department of Orthopedics, Elizabeth-TweeSteden Ziekenhuis, Tilburg, the Netherlands; 4 Department of Medical and Clinical Psychology, Tilburg University, Tilburg, the Netherlands; 5 Department of Orthopedics and Sports Medicine, Erasmus Medisch Centrum, Rotterdam, the Netherlands; 6 OCON Centre for Orthopaedic Surgery and Sports Medicine, Hengelo, the Netherlands; 7 Department of Orthopedics, Onze Lieve Vrouwe Gasthuis, Amsterdam, the Netherlands; 8 Department of Clinical Epidemiology, Leiden University Medical Center, Leiden, the Netherlands; 1 Department of Orthopaedics, Medisch Spectrum Twente, Enschede, the Netherlands; 2 Department of Orthopaedics, Amphia, Breda, the Netherlands

**Keywords:** Expectations, Knee prosthesis, Reoperation, Hip prosthesis, Revision, Partner, Significant other, revision surgeries, JOINt replacement, Hip, Knee, hip and knee arthroplasty, Numeric Rating Scale (NRS), Oxford Hip Score, total hip or knee arthroplasty, patient-reported outcome measures (PROMs), functional outcomes

## Abstract

**Aims:**

The number of arthroplasty revision surgeries is increasing, yet patient-centred outcomes such as mental wellbeing, fulfilment of expectations for postoperative outcomes such as pain, function, and activities of daily living, as outlined in the Hospital for Special Surgery Knee Scale (HSS), and the Hip/Knee Replacement Expectations Survey (HRES/KRES), as well as the importance patients assign to such outcomes, remain insufficiently understood. Moreover, little is known about the role of their significant others. While essential to patients’ recovery and wellbeing, they may experience substantial emotional and practical burdens themselves. Here we present the protocol of the REJOIN study (Outcomes of REvision surgery after primary JOINt replacement), which aims to examine the impact of revision surgery on patients and their significant others. The study describes patients’ expectations and their fulfilment, mental wellbeing, functional outcomes, and the perceived value of outcomes following surgery, as well as the health-related quality of life (QoL) and caregiver burden experienced by their significant others. In addition, it investigates risk factors associated with the aforementioned outcomes.

**Methods:**

This will be a multicentre, longitudinal observational study. All eligible patients scheduled for revision surgery after total hip or knee arthroplasty between March 2026 and February 2033 are invited to participate together with their significant other. We aim to include 3,650 patients. Data will be gathered preoperatively, and at three, six, 12, and 24 months.

**Results:**

The main outcome parameters are expectations and their fulfilment, measured through three different data sources: patient-reported outcome measures (PROMs) gathered for usual care, patients’ medical files, and study questionnaires. The PROMs comprise of the HSS, HRES/KRES, mental health (Pain Catastrophizing Scale (PCS), Hospital Anxiety and Depression Screen (HADS)), QoL (EuroQol five-dimension questionnaire (EQ-5D)), pain (Numeric Rating Scale (NRS) pain), hip/knee functioning (Oxford Hip Score/Oxford Knee Score (OHS/OKS)), patient-prioritized importance of outcomes (their top three), strain on significant other (CarerQol-7D), positive experiences of significant other (PES), and hip/knee function according to significant other (OHS/OKS by significant other).

**Conclusion:**

The REJOIN study offers a systematic approach to evaluate the impact of revision surgery on patients and their significant others with regard to expectation fulfilment, mental wellbeing, and functional outcome.

Cite this article: *Bone Jt Open* 2026;7(11):1439–1445.

## Introduction

Parallel to the globally increasing demand for arthroplasty, concerns have been raised due to an absolute increase in complications as well as the number of revision surgeries.^1^ Major complications of primary hip and knee arthroplasty include infection, instability, aseptic loosening, malalignment, and periprosthetic fracture for which revision surgery is often required.^2-5^ Compared with primary arthroplasty, revision procedures are technically more complex, require more intensive rehabilitation, and carry an increased risk of complications and mortality.^6^ Since the decision to operate depends on the anticipated improvement in quality of life, it requires a comprehensive evaluation not only of patients’ complaints, but also of their psychosocial circumstances and expectations.^7^

Hence, a holistic approach is needed to facilitate well-informed, shared decision-making, as well as the identification of vulnerable patients, so that treatment strategies can be tailored to patients’ needs. The biopsychosocial framework as proposed by Engel^8^ supports this, and states that treatment outcomes are the result of a multifaceted interplay of physical, mental, and social factors. In this regard, studying psychosocial factors of revision surgery is of profound clinical relevance. It is particularly relevant to involve the patients’ significant others in these studies, as they play a pivotal role in the postoperative period while experiencing a substantial emotional and practical burden. However, while extensive research exists on patient expectations and outcomes following primary arthroplasty, current studies provide a limited evidence base for revision surgery, highlighting the need for further investigation.

The Outcomes of REvision surgery after primary JOINt replacement (REJOIN) study primarily aims to examine the impact of revision surgery on patients and their significant others. Firstly, the study describes patients’ expectations and their fulfilment, mental wellbeing, functional outcomes, and the perceived value of outcomes following surgery, as well as the health-related quality of life and caregiver burden experienced by significant others. Secondly, the study investigates risk factors associated with the aforementioned outcomes. Ultimately, this study will help inform decisions over which patients are most likely to benefit from revision surgery.

## Methods

### Study design and setting

The REJOIN study is a multicentre, longitudinal observational study on patients’ expectations and their fulfilment, mental wellbeing, functional outcomes, and the perceived value of these outcomes after total hip or total knee arthroplasty (THA/TKA), as well as the health-related quality of life and caregiver burden experienced by significant others. The study includes patients who are eligible for or had revision surgery after THA or TKA, and their partner or significant other. The inclusion period is five years with active follow-up during two years, thus the overall study duration is seven years. Currently, eight hospitals have agreed to participate: Alrijne Ziekenhuis, Leiderdorp; Amphia Ziekenhuis, Breda; Elisabeth-TweeSteden Ziekenhuis (ETZ), Tilburg; Erasmus Medisch Centrum (EMC), Rotterdam; Leiden University Medical Centre (LUMC), Leiden; Medisch Spectrum Twente (MST), Enschede; Orthopedisch Centrum Oost Nederland (OCON), Hengelo; and Onze Lieve Vrouwe Gasthuis (OLVG), Amsterdam.

### Study patients and eligibility criteria

In order to be eligible to participate in this study, a participant (the patient and, when present, their significant other) must meet all criteria. Patients meet the criteria if: they are scheduled for a minor or major revision surgery after primary THA and TKA (major revision surgery is defined as revision of at least the acetabular or femoral component (hip) or femoral or tibial component (knee)) from March 2026 until February 2033; or they are scheduled for a minor revision (defined as revision of only the inlay and/or femoral head component (hip) or only the insert of the tibial component and/or revision of the patella(r) component (knee) – patellar resurfacing procedures are included as a separate group) during the same period; are able to complete digital questionnaires; and are aged ≥ 18 years. Significant others (when present) are defined as any individual who has, according to the patient, a profound influence on their life or wellbeing such as their partner, spouses, sibling, etc. Significant others must be able to complete digital questionnaires and be aged ≥ 18 years to meet the inclusion criteria. Exclusion criteria were: no informed consent; insufficient Dutch language skills; and physical or mental status prohibiting participation.

### Outcome measures

This study describes outcomes and expectations up to two years after revision surgery. To diminish the strain on patients, data will be retrieved from three different data sources: patient-reported outcome measures (PROMs) gathered for usual care, patients’ medical files, and study questionnaires. Data will be gathered at baseline (i.e. maximum 182 days before surgery for elective indications, and seven to 21 days postoperatively for acute indications, such as infections and fractures) and at three, six, 12, and 24 months’ follow-up. Patients and significant others will independently complete study questionnaires concerning their sociodemographic details, comorbidities, expectations, and mental health. A detailed description of all outcome measures and their timepoints is shown in Tables I – III.

**Table I.** Patient data collection.

Variable	Outcome	Measures	Baseline	3 mths	6 mths	12 mths	24 mths
LROI (usual care)	Quality of life	EQ-5D^9^	X	X	X (knee)	X	X*
	Hip/knee function	OHS/OKS^10,11^	X	X	X (knee)	X	X*
		NRS^12,13^	X	X	X (knee)	X	X*
		Anchor questions hip/knee^14^		X	X (knee)	X	X*
	Patient characteristics	ASA grade^15,16^	X				
		Sociodemographic characteristics and lifestyle†,^5^	X				
Patients’ medical files	Operation	Operation characteristics	X				
		Complications	X	X	X	X	X
	Radiology	Preoperative radiological imaging	X				
Survey	Comorbidities	SCQ^17,18^	X				
	Expectations	HSS-HRES/KRES^19,20^	X	X	X	X	X
	Value assessment	Patient-prioritized importance of outcomes (top 3)	X	X	X	X	X
	Mental health	PCS^21^	X	X	X	X	X
		HADS^22^	X	X	X	X	X

* This questionnaire will be added to the study survey due to a restricted follow-up period of 12 months for usual care patient-reported outcome measures.

† Patients’ medical files will be consulted additionally to complete missing information.

ASA, American Society of Anesthesiologists; EQ-5D, EuroQol five-dimension questionnaire; HADS, Hospital Anxiety and Depression Scale; HSS-HRES/KRES, Hospital for Special Surgery Hip/Knee Scale-Hip/Knee Replacement Expectations Survey; LROI, Landelijke Registratie Orthopedische Interventies; NRS, numeric rating scale; OHS/OKS, Oxford Hip/Knee Score; PCS, Pain Catastrophizing Scale; SCQ, Self-administered Comorbidity Questionnaire.

**Table II.** Survey data collection for partner/significant other.

Outcome	Measures	Baseline	3 mths	6 mths	12 mths	24 mths
Sociodemographic characteristics	Sociodemographic characteristics and lifestyle	X				
Comorbidities	SCQ	X				
Quality of life	CarerQol-7d^23^	X	X	X	X	X
	EQ-5D	X	X	X	X	X
	PES^24^	X	X	X	X	X
Hip/knee function according to partner	OHS/OKS by partner	X	X	X (knee)	X	X
	Anchor questions hip/knee by partner	X	X	X (knee)	X	X

EQ-5D, EuroQol five-dimension questionnaire; NRS, numeric rating scale; OHS/OKS, Oxford Hip/Knee Score; PES, Positive Experiences Scale; SCQ, Self-administered comorbidity Questionnaire.

**Table III.** Outcome measures and their description.

Measures	Description
EQ-5D	The EQ-5D is an instrument designed to derive a single cardinal index for the quality weighting of QALYs from five dimensions of health (mobility, self-care, usual activities, pain, mood). The EQ-5D uses valuations derived with the time tradeoff method from a large general population survey to score the self-reported five-dimension health profile.^9^
OHS/OKS	The OKS and OHS are developed to measure hip and knee pain and function; in this study the validated self-reported Dutch version is used. The OHS and OKS hold 12 items and are developed to assess hip and knee function and pain after total hip and knee arthroplasty.^10,11^
NRS pain at rest and during physical activity	To assess pain, the NRS will be used. Patients will be asked to indicate their pain at rest and during physical activity. The NRS Pain is an 11-point ordinal pain intensity scale anchored at each end by opposite statements, which are ‘no pain’ (0) versus ‘worst possible pain’.^12,13^
Anchor questions	Satisfaction will be measured by anchor questions regarding the change of daily functioning since surgery for the hip. In case of knee surgery two anchor questions are posed, regarding the change of daily functioning since surgery and the change in pain complaints since operation. These questions are answered on a 7-point Likert scale ranging from ‘exceedingly worsened’ to ‘exceedingly improved’.^14^
ASA grade	The fitness of patients before surgery will be assessed using the ASA grade; data on ASA grades will be retrieved using the LROI database. Herein ASA I is classified normal healthy patient; ASA II, patient with mild systemic disease; ASA III, patient with severe systemic disease; ASA IV, patient with severe systemic disease that is a constant threat to life; ASA V, moribund patient who is not expected to survive without the operation; ASA VI, declared brain-dead patient whose organs are being removed for donor purposes.^15,16^
Sociodemographic characteristics and lifestyle	Age; sex; weight and length to calculate BMI; status of living; smoking; indication for revision surgery. Indications will be categorized according to definitions of the LROI.^5^ Additional data and general characteristics of partners/significant others will be gathered using questionnaires. These include: age; sex; status of living; living situation; relation to patient; (voluntary) working status; social support.
Operation characteristics	Operation characteristics like surgical technique, surgical approach, operation time, and blood loss.
Complications	Readmissions, reasons for rerevision or readmission as well as treatment options are scored.
Preoperative radiological imaging	The following preoperative radiological imaging will be gathered from the medical file if available: radiograph (AP), full-length standing radiograph, CT, CT rotation, PET CT, MRI.
SCQ	The SCQ is an instrument to gather information on comorbidities. This questionnaire consists of 12 medical conditions selected on their frequency in general practice and their inclusion in commonly used comorbidity instruments.^17^ The questionnaire contains 13 items with yes or no question about comorbidities, treatment, and impact on daily activities and is validated in the Dutch population.^18^
HSS-HRES/KRES	Patients’ expectations of the outcome of surgery will be measured with a validated Dutch version of the HSS-HRES/KRES. This questionnaire consists of items covering treatment expectancy and rationale credibility.^19,20^ In total 17 items are included in the questionnaire; each expectation is scored on a range of 0 (‘back to normal’) through to 3 (‘small improvement’).
Patient-prioritized importance of outcomes (top 3)	To assess the importance of expectations and pain and function (i.e. the patient-specific level of importance assigned to an expectation or complaint) we established a question in which patients are asked to rank the three most important/valuable expectations or complaints mentioned in the HSS-HRES/KRES and OHS/OKS, in decreasing order of importance. Since this ranking has not been used before, pilot testing of the questionnaire was performed beforehand. The three-step-test-interview is used to qualitatively assess the content validity of the stem question.^25^ This method employs both the think-aloud method, which aims to elucidate the thought process of patients by verbalizing their thoughts, and probing, which aims to elicit reports about their thinking. The interview took approximately 20 minutes and used a topic guide based on the COSMIN criteria (comprehensibility, relevance, acceptability, and feasibility).^26^
PCS	Pain catastrophizing will be measured using the PCS, which consists of 13 items representing three components of pain catastrophizing: rumination, magnification, and helplessness. Items are scored on a scale ranging from 0 (‘not at all’), through to 4 (‘always’), where higher scores represent more pain catastrophizing. The total score ranges between 0 and 52 and is calculated by summing the item scores. Beside depressive symptoms, pain catastrophizing has been associated with central pain sensitization, a finding associated with surgical outcomes.^21^
HADS	Anxiety and depression will be assessed by the HADS, which consists of 14 items divided into a depression and anxiety scale, both consisting of seven items. Items are scored on a scale ranging from 0 to 3, with higher scores indicating more complaints. A total score of 0 to 7: no depression/anxiety disorder; 8 to 10: a ‘possible’ depression/anxiety disorder; 11 to 21: ‘suspected’ depression/anxiety disorder.^22^
CarerQol-7D	The CarerQol-7D is designed to measure and value the impact of providing informal care on caregivers. It consists of seven items that are scored on a scale from 0 (‘no’) through to 2 (‘a lot’). The questionnaire concludes with a visual analogue scale ranging from 0 (‘completely unhappy’) to 10 (‘completely happy’). The weighted sum is calculated using a tariff accounting for the severity of problems.^23^
PES	To measure positive experiences by partners/significant others, the PES was used.^24^ This questionnaire utilizes a unidimensional hierarchical Mokkenscale which varies from intrinsic satisfaction and relational enhancement to improvement of competence and social enhancement. It provides for a useful questionnaire due to its psychometric qualities and its broad use in different populations of informal caregivers. In total eight items are scored on a scale from 0 (‘agree’) through to 2 (‘disagree’).
OHS/OKS by partner	The questionnaire is identical to the one distributed to the patient, except for a changed perspective. Partners/significant others of patients are asked to fill in the OHS/OKS for the patients according to themselves.^10,11^
Anchor questions by partner	Questions are identical to the ones distributed to the patient. Partner/significant other are asked to rate the change of daily functioning since surgery in day-to-day life on a 7-point Likert scale ranging from ‘exceedingly worsened’ to ‘exceedingly improved’.^14^

AP, anteroposterior; ASA, American Society of Anesthesiologists; EQ-5D, EuroQol five-dimension questionnaire; HADS, Hospital Anxiety and Depression Scale; HSS-HRES/KRES, Hospital for Special Surgery Hip/Knee Replacement Expectations Surveys; LROI, Landelijke Registratie Orthopedische Interventies; NRS, numeric rating scale; OHS, Oxford Hip Score; OKS, Oxford Knee Score; PCS, Pain Catastrophizing Scale; PES, Positive Experiences Scale; PET, positron emission tomography; QALY, quality-adjusted life-year; SCQ, Self-administered Comorbidity Questionnaire.

### Sample size and power considerations

This observational study is designed to continuously include patients scheduled for revision surgery of THA or TKA, and will thus provide an ongoing base for future studies. This study is comparable to the Longitudinal Leiden Orthopedics Outcomes of OsteoArthritis Study (LOAS) a former ten-year observational study on primary hip, knee, and shoulder arthroplasty.^27^ According to hospital records of Alrijne Ziekenhuis, Amphia Ziekenhuis, ETZ, EMC, LUMC, MST, OCON, and OLVG, a total of 575 hip and 475 knee revisions are performed annually. Based on previous experience with a comparable observational cohort of primary THA and TKA patients, we anticipate that approximately 70% of patients and significant others will consent to participate in this study.^12^ As such, we aim to include approximately 400 hip and 325 knee revision patients within the first year. With these samples we will be able to prove correlations of 0.15 (p < 0.05, power 80%). In the following four years we expect to include another 2,900 patients, leading to an accrual of 3,650 patients.

### Statistical analysis

The questionnaires will gather both continuous and categorical data. All data will be entered and stored in one database. Data analysis will be carried out using SPSS v. 29.0.0.0 (IBM, USA) or R v. 4.5.1 (R Foundation for Statistical Computing, Austria). To describe the patient characteristics and primary outcomes, descriptive statistics will be used. Data will be stratified for hip and knee joints and corresponding indication for revision. Trends over time will be modelled with multivariate analyses (repeated measures analysis). With regard to investigating risk factors associated with the aforementioned outcomes, appropriate multivariate regression analysis will be used, e.g. mixed model analysis. If possible, similar methods (i.e. mixed models) will be employed to account for possible missing data; when this is not possible, multiple imputation will be performed. When appropriate, statistical analyses will be corrected for confounding factors identified by current literature.

### Recruitment and data collection

Healthcare professionals in the participating hospitals will inform eligible patients about the study. At baseline, eligible patients will receive an email containing a link to the informed consent form. After informed consent is obtained, patients will be guided through the standardized data capturing system where the questionnaires are provided. Reminder messages will be issued if a patient does not respond after the questionnaire has been sent. Patients who decline further participation will be recorded as dropouts.

### Ethics, data management, funding, and disclosures

Due to the limited strain on patients and the absence of further alterations of usual care, this study was deemed exempt from ethical approval by the non-WMO committee of the LUMC on 16 December 2025. It will be conducted according to the principles of the 2024 Declaration of Helsinki,^28^ and in accordance with national and institutional guidelines, regulations, and acts. The REJOIN study is funded by the orthopaedic department of the LUMC. We gratefully acknowledge the additional support provided by the other hospitals participating in this consortium: Alrijne ziekenhuis, Amphia ziekenhuis, ETZ, EMC, MST, OCON and OLVG. Authors of this article are members of the steering committee of the REJOIN study.

## Discussion

Revision arthroplasty surgery is associated with significantly higher complication rates and a more demanding rehabilitation period compared with primary procedures.^6^ Although outcomes of revision surgery have improved, substantial variability in results persist, particularly in elective cases. This variability underscores the need to understand patient expectations, the extent to which these expectations are fulfilled, and the relative importance patients assign to specific outcomes.

In this context, preoperative decision-making should extend beyond joint-specific factors and incorporate a biopsychosocial perspective. Literature indicates that psychological and social factors significantly influence surgical outcomes, suggesting these may represent additional avenues for holistic treatment.^29^ In addition, outcomes after revision surgery are embedded within a broader social context: the wellbeing and burden of significant others may both influence and be influenced by the patient’s recovery trajectory. However, this interaction remains insufficiently understood.

The REJOIN study is designed to systematically assess psychosocial factors, expectations, their fulfilment, and the perceived importance of outcomes following revision surgery in patients while including the perspective of their significant others. By integrating these aspects within a longitudinal framework, the study aims to provide a more comprehensive understanding of recovery trajectories. In particular, it will identify which factors are associated with functional recovery and health-related quality of life. Consequently, the study aims to support more well-informed, shared decision-making, enable early identification of vulnerable patients, and facilitate treatment strategies.

To our knowledge, this is the largest multicentre study including outcomes from a biopsychological perspective after arthroplasty revision surgery. The REJOIN study constitutes eight hospitals in total, including two university hospitals which enhance the generalizability of the findings. Furthermore, it combines functional outcomes with psychosocial factors, patients’ expectations, their fulfilment, and the relative importance assigned to different outcome domains throughout the entire postoperative period of 24 months, allowing for a more comprehensive evaluation of the recovery trajectory. In addition, the inclusion of significant others provides insight into how patient outcomes and caregiver burden may interact over time. Importantly, the REJOIN study should be viewed as a discovery cohort for future studies.

Several limitations should also be acknowledged. First, the vast heterogeneity in indications for revision surgery may complicate the interpretation of outcomes. Second, although digital questionnaires expand the reach of the study, it may exclude individuals with limited digital literacy, or visual or motor impairments, thus restricting generalizability to the target population. Third, the logistic complexity of the study may provide organizational challenges. To counteract these issues, regular study meetings with local research teams will be held. In addition, a steering committee has been formed to support decision-making throughout the study period.

Currently, there is a paucity of knowledge of how psychosocial factors, expectations, and their fulfilment interact to influence outcomes after revision arthroplasty. By incorporating the perspectives of both patients and their significant other, the REJOIN study provides a unique opportunity to explore these relationships.

As a discovery cohort, this study intends to generate hypotheses regarding the mechanisms underlying variability in recovery, including the role of expectation-outcome mismatches, the relative importance patients assign to different functional domains, and the influence on interactions between patients and their significant others. These insights may inform the development of targeted interventions, such as preoperative expectation management, psychosocial screening tools, and support strategies involving significant others.

In addition, the findings may contribute to improved patient selection and shared decision-making by identifying profiles of patients and support systems associated with favourable or unfavourable outcomes.

**Take home message**

- A discovery cohort intended to generate hypotheses regarding the mechanisms underlying variability in recovery, including the role of expectation-outcome mismatches, the relative importance patients assign to different functional domains, and the influence on the interactions between patients and their significant other interactions.

- These insights contribute to improved patient selection and shared decision-making, and may inform the development of targeted interventions.

## Data Availability

The data that support the findings for this study are available to other researchers from the corresponding author upon reasonable request.
